# Genetic Platforms of *bla*_CTX-M_ in Carbapenemase-Producing Strains of *K. pneumoniae* Isolated in Chile

**DOI:** 10.3389/fmicb.2018.00324

**Published:** 2018-03-06

**Authors:** Sergio Carrasco-Anabalón, Alejandra Vera-Leiva, Mario Quezada-Aguiluz, María F. Morales-Rivera, Celia A. Lima, Jorge Fernández, Soledad Ulloa, Mariana Domínguez, Gerardo González-Rocha, Helia Bello-Toledo

**Affiliations:** ^1^Laboratorio de Investigación en Agentes Antibacterianos, Departamento de Microbiología, Universidad de Concepción, Facultad de Ciencias Biológicas, Concepción, Chile; ^2^Laboratorio Central, Hospital Regional Dr. Guillermo Grant Benavente, Concepción, Chile; ^3^Laboratorio Biomédico Nacional, Instituto de Salud Pública de Chile, Santiago, Chile

**Keywords:** genetic platforms, *bla*_**CTX-M**_, *Klebsiella pneumoniae*, KPC, OXA-370, NDM-1, MDR

## Abstract

**Objective:** To elucidate whether the genetic platforms of *bla*_CTX-M_ contribute to the phenotypes of multi-drug-resistance (MDR) in the first carbapenemase-producing *K. pneumoniae* strains isolated in Chile.

**Method:** Twenty-two carbapenemase-producing *K. pneumoniae* strains isolated from different Chilean patients and hospitals were studied. Their genetic relatedness was assessed by PFGE and MLST. The levels of antibiotic resistance were evaluated by determining the minimum inhibitory concentration of various antimicrobials. In addition, several antibiotic resistance genes of clinical relevance in Chile were investigated. The prevalence, allelic variants, and genetic platforms of *bla*_CTX-M_ were determined by PCR and sequencing.

**Results:** Out of the 22 strains studied, 20 carry KPC, one carries NDM-1, and one carries OXA-370. The PFGE analysis showed three clades with a genetic relatedness >85%, two formed by four strains and one by eight strains. The other strains are not genetically related, and a total of 17 different pulse types were detected. Ten different STs were identified, the main ones being ST258 (five strains) and ST1161 (seven strains). The isolates presented different percentages of resistance, and 82% were resistant to all the β-lactams tested, 91% to ciprofloxacin, 73% to colistin, 59% to gentamicin, 50% to amikacin, and only 9% to tigecycline. All isolates carried *bla*_TEM_ and *bla*_SHV_, whereas 71% carried *aac(6*′*)Ib-cr*, and 57% one *qnr* gene (*A, B, C, D*, or *S*). The *bla*_CTX-M_ gene was found in 10 of the isolates (4 *bla*_CTX-M−15_ and 6 *bla*_CTX-M−2_). The characterization of the platform, in seven selected strains, revealed that the gene is associated with unusual class 1 integrons and insertion sequences such as IS*CR1*, IS*ECp1*, and IS*26*.

**Conclusion:** In the first carbapenemase-producing *K. pneumoniae* strains isolated in Chile the genetic platform of *bla*_CTX-M−2_ corresponds to an unusual class 1 integron that can be responsible for the MDR phenotype, whereas the genetic platforms of *bla*_CTX-M−15_ are associated with different IS and do not contribute to multi-drug resistance.

## Introduction

*Enterobacteriaceae* resistant to third-generation cephalosporins, carbapenems, or both, are one of the critical priorities and represent one of the greatest challenges in the epidemiology of antibiotic resistance (Guzmán-Blanco et al., 2014; Barría-Loaiza et al., 2016; Liu et al., 2016; WHO, 2017). According to reports, some countries in the Americas, such as Argentina, Brazil, Colombia, Puerto Rico, and the United States have endemic strains of carbapenemase-producing *Enterobacteriaceae* (Lee et al., 2016).

A surveillance program on carbapenemase-producing *Enterobacteriaceae* has been implemented in Chile and the first isolation occurred in 2012 (Cifuentes et al., 2012). Since then, and up to 2015, only 34 isolates were reported (ISP Chile, 2015), which would suggest good healthcare infection control practices. However in the same period, the incidence of *K. pneumoniae* strains resistant to third generation cephalosporins was around 70% (ISP Chile, 2015), and in up to 60% of the isolates such resistance was mediated by extended-spectrum β-lactamases (ESBLs) (Guzmán-Blanco et al., 2014).

CTX-M enzymes are among the most important ESBLs in the world, with a clear higher prevalence than other ESBLs, such as TEM-, SHV-, GES-, and PER-type (Bello et al., 2005; García et al., 2011; Cantón et al., 2012b; Wozniak et al., 2012). The successful spread of CTX-M is determined by multiple factors, including the genetic platforms of the *bla*_CTX-M_ gene. Different architectures have been identified in such platforms, and roughly two fundamental elements are recognized. On one hand, the platforms can be composed of integrons, which promote multi-drug-resistance (MDR) phenotypes, namely non-susceptibility to at least one agent in three or more antimicrobial categories (Magiorakos et al., 2012). On the other hand, the presence of insertion sequences, such as IS*CR1* or IS*Ecp1*, act as promoters for the expression of various resistance genes and influence the mobilization of the *bla*_CTX-M_ genes (Power et al., 2005; Cantón et al., 2012b).

In general, the production of CTX-M enzymes is associated with MDR profiles, involving mainly resistance to third-generation cephalosporins, quinolones, aminoglycosides, and trimethoprim (Cantón et al., 2012b). In turn, the frequent association of other resistance genes, such as *aac(6*′*)Ib-cr* and *qnr* genes, with the successful dissemination of CTX-M enzymes in *K*. *pneumoniae* strains has been reported (Sabtcheva et al., 2009; Elgorriaga-Islas et al., 2012; Bado et al., 2016). This has led to an increase in the clinical usage of carbapenems, creating a selective pressure on resistant strains (Cantón et al., 2012a; Falagas et al., 2014; Cifuentes et al., 2015).

In Chile the production of CTX-M is the main mechanism of resistance to third-generation cepahalosporins in *K. pneumoniae* (Cifuentes et al., 2012, 2015), even in carbapenemase-producers. Nevertheless, there is no information about the genetic surroundings of *bla*_CTX-M_ and its contribution to the MDR phenotype in carbapenemase-producing isolates. Thus, the aim of this study was to determine the genetic platforms associated with *bla*_CTX-M_, and their contribution to the MDR phenotype observed in the first carbapenemase-producing *K. pneumoniae* strains isolated during a period of 3 years in Chilean hospitals.

## Materials and methods

### Strains

**Figure 1:**
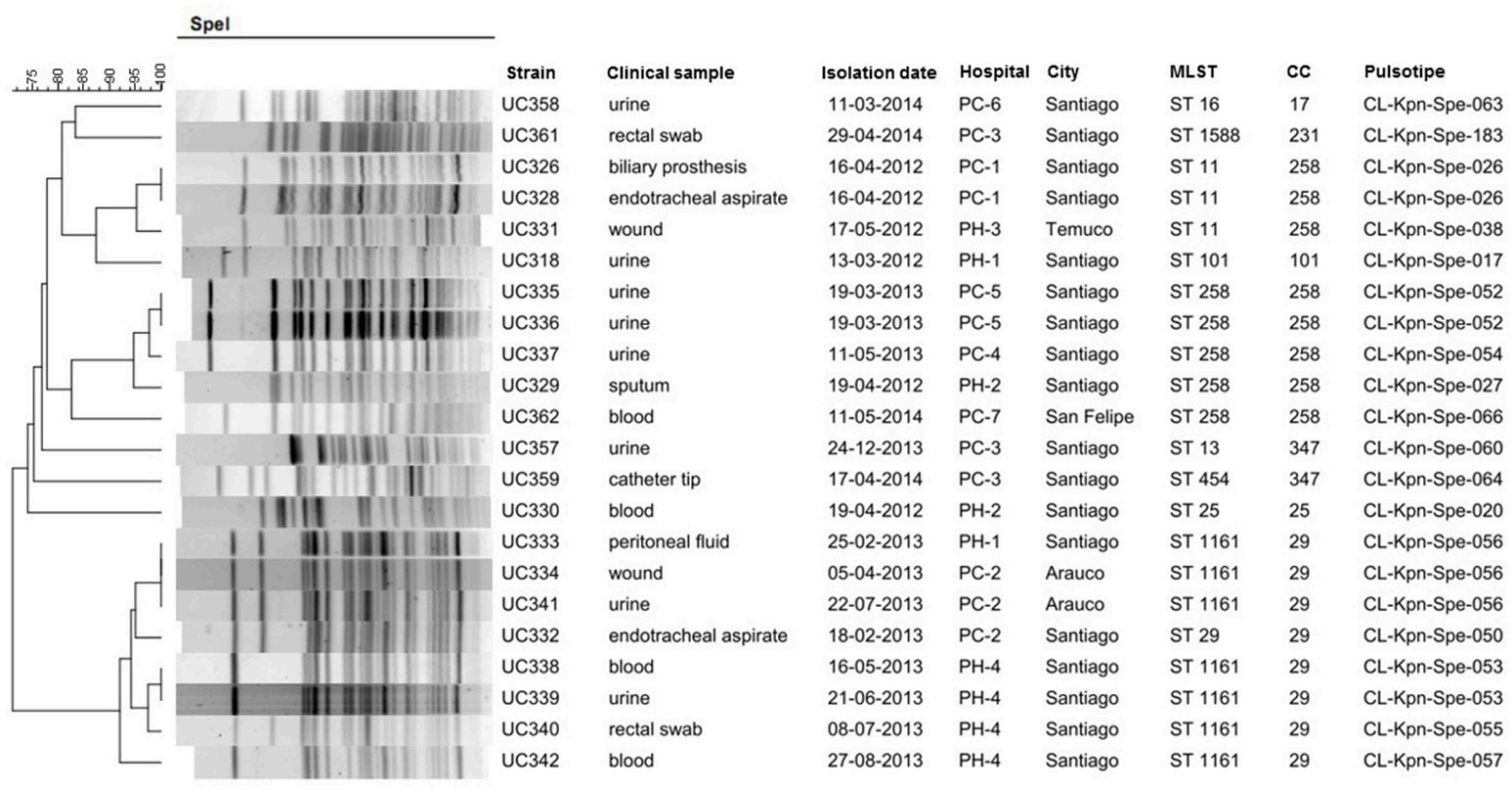
Genetic relatedness origin and molecular characteristics of carbapenemase-producing *Klebsiella pneumoniae* isolated in Chilean hospitals. PC, private clinic; PH, public hospital; MLST, multilocus sequence typing; ST, sequence type; CC, clonal complexes.

All first 22 carbapenemase-producing *K. pneumoniae* strains isolated from public (PH) and private (PC) Chilean hospitals between 2012 and 2014 were included. Eighteen strains were isolated in Santiago (the capital of Chile), one in San Felipe (90 Km north of Santiago), two in Arauco (570 Km south of Santiago), and one in Temuco (680 Km south of Santiago), and were isolated from different patients. The Chilean Public Health Institute [Instituto de Salud Pública (ISP), Santiago, Chile] provided the strains as part of the surveillance program of carbapenemases in *Enterobacteriaceae*. The origin and molecular characteristics of the isolates are shown in Figure 1.

### Pulsed-field gel electrophoresis (PFGE)

Bacterial DNA was prepared and digested with 50 U of *SpeI* endonuclease (Thermo Fisher Scientific Inc., Waltham, MA) as previously described (Woodford et al., 2004). The PFGE patterns were analyzed with BioNumerics software v6.6 (Applied Maths) by using the Dice coefficient. The dendogram was constructed according to the unweighted pair group method with arithmetic mean (UPGMA). Tolerance and optimization parameters were set to 1.5% each. Isolates with ≥ 85% similarity were considered genetically related (Giakkoupi et al., 2011).

### Multilocus sequence typing (MLST)

For MLST, seven housekeeping genes (*gapA, infB, mdh, pgi, phoE, rpoB*, and *tonB*) were amplified and sequenced according to the protocol described for *K. pneumoniae* on the Institut Pasteur MLST databases^1^.

### Antimicrobial susceptibility testing

Minimum inhibitory concentrations (MICs) were determined by the agar dilution method according to recommendations and breakpoints proposed by the CLSI (CLSI, 2017). The antibiotics assayed were imipenem (IPM), ertapenem (ETP) (Merck Sharp & Dohme Corp., Kenilworth, NJ, USA), meropenem (MER) (Sigma-Aldrich, St Louis, MO, USA), ceftazidime (CAZ), cefotaxime (CTX), ciprofloxacin (CIP), gentamicin (GEN), and amikacin (AMK) (Merck Sharp & Dohme Corp., Kenilworth, NJ, USA). MICs of colistin (COL) (Sigma-Aldrich) and tigecycline (TIG) (Pfizer, Philadelphia, PA, USA) were determined by the broth microdilution method, using the European Committee on Antimicrobial Susceptibility Testing (EUCAST, 2017) guidelines and break-points since breakpoints for these two antibiotics are not supplied by the CLSI.

### Detection of resistance genes

Total DNA was extracted using the commercial kit Instagene Matrix^TM^ (BIO-RAD, Hercules, CA, USA) according to manufacturer recommendations. PCR assays were used to detect the β-lactamase-encoding genes *bla*_TEM_, *bla*_SHV_, *bla*_CTX-M_ (Sánchez et al., 2006; Woodford et al., 2006; Geser et al., 2012). The PCR products were sequenced (Macrogen, Seoul, Korea) and the nucleotide sequences and their derived amino acid sequences were compared to the existing sequences in the GenBank database (National Center for Biotechnology Information, NCBI) and in the Lahey β-lactamase classification database^2^ using the BLAST^3^, and ExPASy translate tools^4^. Sequences were aligned using Clustal-Omega software^5^. Additional genes of antibiotic resistance, such as plasmid-mediated quinolone resistance (PMQR) genes [q*nrA, qnrB, qnrS, qnrC, qnrD, aac(6*′*)Ib-cr;* (Chen et al., 2012)], were included in order to further characterize the strains. In all strains, the carbapenemase gene was confirmed by PCR. All the primers are listed in Table S1.

### Determination of the genetic environment of *bla*_CTX-M_

**Figure 2:**
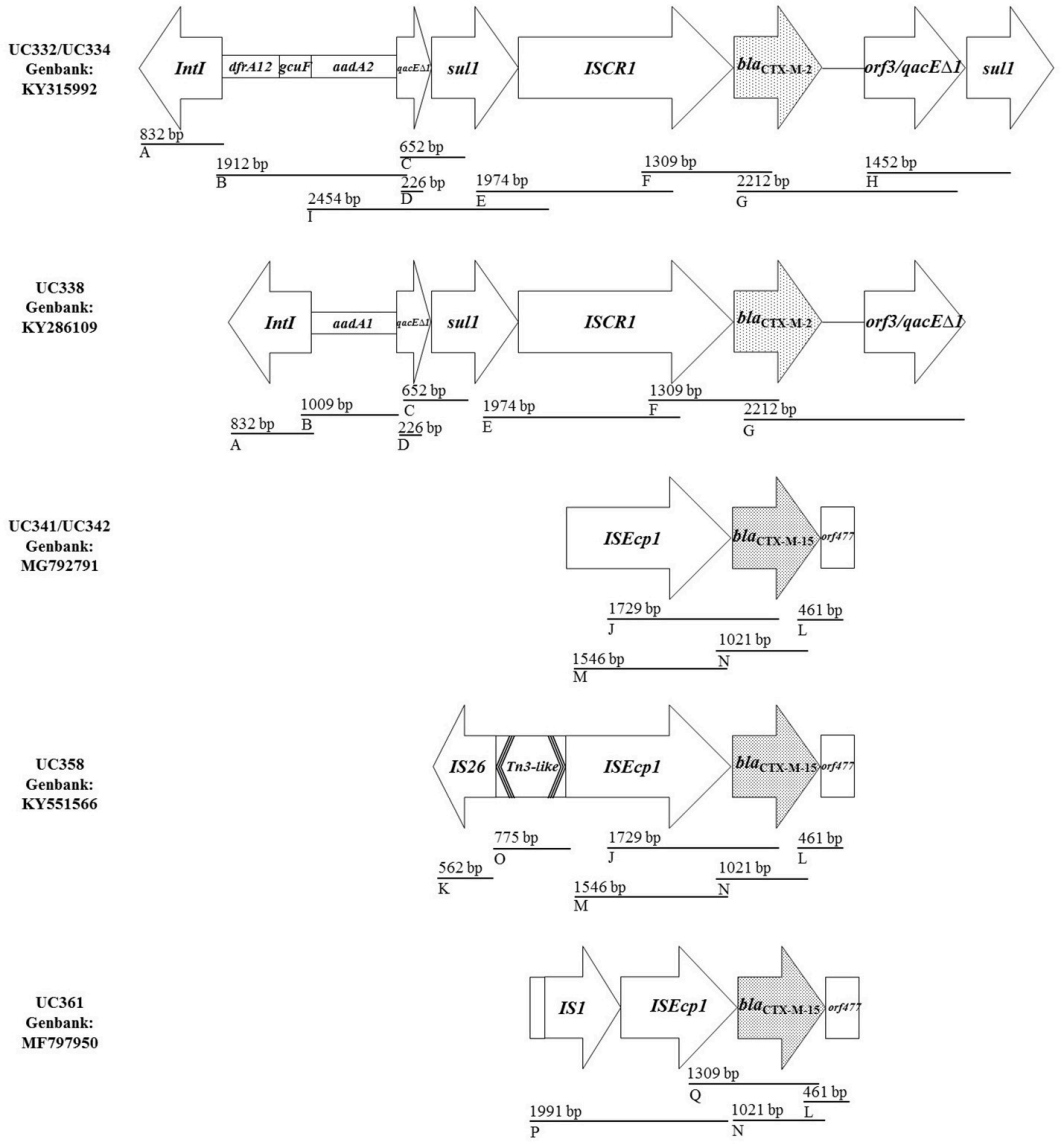
Genetic platforms of *bla*_CTX-M_ in carbapenemase-producing *Klebsiella pneumoniae* isolated in Chilean hospitals. Thick lines as well as the lengths (bp) of the PCR-products obtained indicate the strategy used for PCR mapping. The letters at the bottom left of each line indicate the different PCR primer pairs used (the primers were listed in Tables S1–S4).

The characterization was performed on seven isolates selected according to the following criteria: different allelic variant of *bla*_CTX-M_, carbapenemase type, pulse type, ST, and city of origin. The genetic environment of *bla*_CTX-M_ was investigated by PCR-mapping of the regions upstream and downstream of the gene, using previously described references (Gaze et al., 2005; Power et al., 2005; Eckert et al., 2006; Vignoli et al., 2006). The strategy is shown in Figure 2, and the primers used are listed in Tables S1–S4. The PCR-products were sequenced (Macrogen, Seoul, Korea) and the resulting sequences were assembled using CAP3 software^6^.

## Results

### Molecular typing

Molecular typing by PFGE showed 17 pulse types arranged in three clades with >85% genetic similarity. Two clades contain four strains each, and the other eight strains and the remaining strains were not genetically related. Four clonal pulse types were found to be associated with more than one isolate each: CL-Kpn-Spe-026 (UC326/UC328), CL-Kpn-Spe-052 (UC335/UC336), CL-Kpn-Spe-056 (UC333/UC334/UC341), and CL-Kpn-Spe-053 (UC338/UC339) (Figure 1). Ten different STs were identified; the most common were ST1161 with seven isolates and ST258 with five isolates. Seven clonal complexes (CC) were identified, and CC258 and CC29 were the most prevalent with eight isolates each (Figure 1).

### Antimicrobial resistance

All isolates were found to be resistant to CTX, CAZ, and ETP and only four were susceptible to at least one of the carbapenems assayed (Table 1). Furthermore, 20 isolates were highly resistant to CIP (MIC_50_: 64 mg/L, MIC_90_: 128 mg/L), and 11 and 13 isolates were found to be resistant to AMK and GEN, respectively. Regarding TIG, two isolates were found to be resistant to this antibiotic. The MIC of colistin was >2 mg/L for 16 isolates, thus classified as resistant according to EUCAST guidelines (EUCAST, 2017).

**Table 1:** Resistance features of carbapenemase-producing *K. pneumoniae* spp. isolated in Chilean hospitals.

**Strain**	**Resistance genes**	**MIC (mg/L)**									
		**CTX**	**CAZ**	**ETP**	**IPM**	**MER**	**AMK**	**GEN**	**CIP**	**COL**	**TIG**
UC318	*bla*_KPC-2_, *bla*_SHV-1_, *bla*_TEM-1_	>512	128	512	64	64	512	128	128	4	1
UC326	*bla*_KPC-2_, *bla*_SHV-1_, *bla*_TEM-1_,*aac(6′)Ib-cr, qnrB*	>512	64	64	32	16	8	16	64	4	4
UC328	*bla*_KPC-2_, *bla*_SHV-1_, *bla*_TEM-1_,*aac(6′)Ib-cr, qnrB*	64	32	512	128	64	64	64	64	4	4
UC329	*bla*_KPC-2_, *bla*_SHV-1_, *bla*_TEM-1_, *aac(6′)Ib-cr, qnrB*	64	128	32	8	4	32	2	128	4	1
UC330	*bla*_KPC-2_, *bla*_SHV-1_, *bla*_TEM-1_, *aac(6′)Ib-cr, qnrB*	32	128	64	8	16	32	2	128	2	1
UC331	*bla*_KPC-24_, *bla*_SHV-1_, *bla*_TEM-1_	64	64	32	4	4	32	1	128	8	1
UC332	*bla*_KPC-2_, *bla*_SHV-1_, *bla*_TEM-1_, *bla*_CTX-M-2_, *aac(6′)Ib-cr*	>512	64	128	8	32	>512	>512	64	32	≤0.5
UC333	*bla*_KPC-2_, *bla*_SHV-1_, *bla*_TEM-1_, *bla*_CTX-M-2_,*aac(6′)Ib-cr, qnrB, qnrD*	>512	256	512	16	32	>512	>512	64	16	1
UC334	*bla*_KPC-2_, *bla*_SHV-1_, *bla*_TEM-1_, *bla*_CTX-M-2_, *aac(6′)Ib-cr, qnrB, qnrD*	>512	32	256	64	64	>512	>512	64	16	0,5
UC335	*bla*_KPC-2_, *bla*_SHV-1/12^*^_, *bla*_TEM-1_, *aac(6′)Ib-cr, qnrB*	512	512	32	4	4	64	64	128	4	1
UC336	*bla*_KPC-2_, *bla*_SHV-1/12^*^_, *bla*_TEM-1_, *aac(6′)Ib-cr, qnrB*	128	>512	64	8	4	64	64	128	4	1
UC337	*bla*_KPC-2_, *bla*_SHV^*^^†^_, *bla*_TEM-1_, *aac(6′)Ib-cr*	64	128	32	8	4	64	64	128	4	1
UC338	*bla*_KPC-2_, *bla*_SHV-1_, *bla*_TEM-1_, *bla*_CTX-M-2_, *aac(6′)Ib-cr*	>512	128	128	16	32	>512	1	64	32	≤0.5
UC339	*bla*_KPC-2_, *bla*_SHV-1_, *bla*_TEM-1_, *bla*_CTX-M-2_	64	32	256	8	32	2	1	64	32	≤0.5
UC340	*bla*_KPC-2_, *bla*_SHV-1_, *bla*_TEM-1_, *bla*_CTX-M-2_, *qnrB, qnrS*	>512	512	128	2	4	4	1	64	4	≤0.5
UC341	*bla*_KPC-2_, *bla*_SHV-1_, *bla*_TEM-1_, *bla*_CTX-M-15_, *aac(6′)Ib-cr*	>512	32	64	8	32	>512	>512	64	16	≤0.5
UC342	*bla*_KPC-2_, *bla*_SHV-1_, *bla*_TEM-1_, *bla*_CTX-M-15_, *aac(6′)Ib-cr*	32	64	128	8	32	32	16	64	32	≤0.5
UC357	*bla*_KPC-2_, *bla*_SHV-1_, *bla*_TEM^†^_, *aac(6′)Ib, qnrB*	64	32	8	2	4	4	8	32	≤0,5	≤0.5
UC358	*bla*_OXA-370_, *bla*_SHV^†^_, *bla*_TEM^†^_, *bla*_CTX-M-15_, *aac(6′)Ib, qnrB, qnrS*	>512	>512	8	2	4	≤2	1	128	≤0,5	2
UC359	*bla*_KPC-2_, *bla*_SHV^†^_, *bla*_TEM-1_, *aac(6′)Ib, qnrB, qnrS*	8	32	2	≤0.5	≤0,5	8	1	2	≤0.5	≤0.5
UC361	*bla*_NDM-1_, *bla*_SHV^*^^†^_, *bla*_TEM^†^_, *bla*_CTX-M-15_,*aac(6′)Ib-cr, qnrB, qnrC*	512	>512	8	16	16	≤2	64	2	≤0.5	≤0.5
UC362	*bla*_KPC-2_, *bla*_SHV^†^_, *bla*_TEM-1_, *aac(6′)Ib-cr*	256	256	64	16	64	64	64	64	≤0.5	2

* *SHV BLEE-type by RFLP*.

† *Variant not elucidated*.

### Identification of antibiotic resistance genes

The *bla*_KPC_ gene was identified in 20 *K. pneumoniae* strains (19 *bla*_KPC−2_ and one *bla*_KPC−24_), *bla*_NDM−1_ in _one_ strain, and *bla*_OXA−370_ in another_._ The gene *bla*_CTX-M_ was detected in 10 isolates, in four it corresponds to the allele *bla*_CTX-M−15_ and in six to *bla*_CTX-M−2_ (Table 1). All isolates carry the gene *bla*_SHV_, and in four it corresponds to an ESBL variant of the enzyme. All isolates also carry *bla*_TEM_, 19 of which have the *bla*_TEM−1_ allele, and the variant was not elucidate in the other three isolates. The resistance genes for other antibiotics included three isolates with *aac(6*′*)-Ib* and 15 with *aac(6*′*)-Ib-cr* variant. Regarding the *qnr* gene, 13 isolates carry *qnrB*, and five carry other variants such as *qnrD, qnr*S, or *qnrC* (Table 1).

### Characterization of the *bla*_CTX-M_ genetic context

Seven isolates were selected for characterization of the *bla*_CTX-M_ genetic context: UC332, UC334, UC338, UC341, UC342, UC358, and UC361. In the isolates carrying the *bla*_CTX-M−2_ variant (UC332, UC334, UC338), the gene was found immediately next to the IS*CR1* genetic element, forming part of a complex class 1 integron (Figure 2). The genetic platforms of the isolates UC332/UC334 shared 100% amino acid similarity (Genbank: KY315992). Also, the variable region (1,912 bp) of the complex class 1 integron was composed by the gene cassettes *dfrA12, gcuF* and *aadA2* in both of these strains (Figure 2); while, the variable zone of isolate UC338 (1,009 bp) comprised the cassette *aadA1*. Unlike in UC332/UC334, the *sul1* gene was not observed in the duplication of the extreme 3′CS of the complex class 1 integron of strain UC338 (Figure 2).

In addition, the isolate UC332 carried another class 1 integron (not associated with *bla*_CTX-M−2_), and its variable zone of 4100 bp consisted of the gene cassettes *arr-2, cmlA5, bla*_OXA−10_, and *aadA1* (Genbank accession number: MF113045).

In isolates bearing the *bla*_CTX-M−15_ variant (UC341, UC342, UC358, and UC361) three platforms were found (Figure 2). In all of them the gene was found to be flanked upstream by the IS*Ecp1* insertion sequence, and downstream by the open reading frame of the hypothetical protein Δ*orf477*. The genetic platforms of the isolates UC341/UC342 shared 100% of DNA similarity (Genbank: MG792791), but none was amplified upstream of IS*Ecp1* with primers used, as occurred with the platform of isolate UC358 and UC361. The platform of UC358 (Genbank accession number: KY551566) corresponds to a nucleotide sequence similar to a section of transposon Tn*3*, previously named Tn*3*-like (Genbank accession number: AB976579). This region preserves the 3′−5′ sense coding regions of the resolvase (*tnpR*) and the 5′−3′ sense-coding portion of the transposase (*tnpA*) found in the Tn*3* family. However, both genes are truncated: *tnpR* by the IS*26* insertion sequence and *tnpA* by IS*Ecp1* (Figure 2).

On the other hand, in strain UC361 (Genbank accession number: MF797950), IS*Ecp1* was truncated by the IS*1* insertion sequence; it retained the promoter region of the *bla*_CTX-M−15_ gene, but the *tnpA* promoter region of IS*Ecp1* was absent. The insertion of IS*1* resulted in the displacement of part of nucleotide sequence IS*Ecp1* toward the 5′ end of the platform (Figure 2).

## Discussion

The MDR phenotype reported in all studied isolates is frequently associated with CTX-M and carbapenemase-producing strains (Cantón et al., 2012b; Geser et al., 2012; Guzmán-Blanco et al., 2014). Like elsewhere in the world (Woodford et al., 2004; Falagas et al., 2014; Pereira et al., 2015), KPC is the most prevalent carbapenemase present in *K. pneumoniae* in Chile (Vera-Leiva et al., 2017). Nevertheless, the description of NDM-1 and OXA-370 in two isolates included in this study is epidemiologically of note, because they represent the first report of these carbapenemases in Chile. The pulse types of these two strains have a genetic identity < 85% with respect to the KPC producing strains, as shown in Figure 1. Also, their STs have only been previously described in Brazil, in strains producing the same carbapenemases; furthermore the OXA-370 variant had been only reported previously in Brazil (Pereira et al., 2015; Aires et al., 2017). This leads us to hypothesize that both isolates could represent cases imported from Brazil.

The characterization of the *bla*_CTX-M_ genetic platforms in the present work is one of the few reports in the literature in South America, complementing previously reported work in Argentina and Uruguay regarding *bla*_CTX-M−2_ where the gene was also found to be associated with IS*CR1* forming part of complex or unusual class 1 integrons (Power et al., 2005; Vignoli et al., 2006). Nucleotide sequences similar to the complete *bla*_CTX-M−2_ platforms in the isolates UC332 and UC334 have been previously reported elsewhere, such as in French Guiana in 2004 (Genbank: EF592571), Uruguay in 2005 (Genbank: EU780013) and in the United States in 2012 (Genbank: KU254578). This suggests the platform may be widely disseminated globally. No arrangement similar to the complete genetic platform found in strain UC338 (Genbank: KY286109) has been previously reported in the NCBI nucleotide database.

The diversity of the characterized genetic platforms of *bla*_CTX-M−15_ was greater than for the *bla*_CTX-M−2_, reflecting a complex scenario associated with insertion sequences that can act as mobilization and expression tools for various β-lactamase genes (Eckert et al., 2006). In strains UC341 and UC342 (Figure 2), the *bla*_CTX-M−15_ platform corresponds to the most frequently described structure in several geographical areas of the world (Eckert et al., 2006; Cantón et al., 2012b). IS*Ecp1* has been described as the insertion sequence responsible for the capture, mobilization and expression of the *bla*_CTX-M_ gene (Poirel et al., 2005); likewise, *orf477*, which is found downstream of *bla*_CTX-M_, has also been frequently described associated with the *bla*_CTX-M_ genes (Cantón et al., 2012b).

The presence of other insertion sequences such as IS*26* or IS*1* upstream of *bla*_CTX-M_ could provide high mobility to this platform and the ability to integrate into the bacterial chromosome, favoring the stability and dissemination of *bla*_CTX-M_ genes (Cantón et al., 2012b).

Using BLAST, two nucleotide sequences with 100% similarity to the *bla*_CTX-M−15_ platform present in the UC358 isolate were found, both associated with *E. coli* ST131, one from Japan in 2011 (Genbank: AB976579) and another from Saudi Arabia 2014 (Genbank: CP015086; Matsumura et al., 2015). The only previous description of a platform with a similar architecture to that of *bla*_CTX-M−15_ present in the UC361 isolate was in *E. coli* isolated in Japan in 2011, but associated with the *bla*_CTX-M−3_ allele instead (Matsumura et al., 2015). This suggests this is a rare genetic platform, unlike those associated with *bla*_CTX-M−2_, and that may be present in different bacterial genera.

This illustrates the high variability among the genetic platforms found. It should be noted that no additional antibiotic resistance genes were found adjacent to *bla*_CTX-M_ in the platform, and as such the platform itself would not be contributing further to the observed multi-resistance phenotypes. However it is interesting to note that *bla*_OXA−370_, which has evolved from *bla*_OXA−48_, results in high levels of resistance to imipenem, but does not affect the susceptibility to broad-spectrum cephalosporins (Poirel et al., 2010). As such, the resistance to this antibiotic class would be due to *bla*_CTX-M−15_ in the UC358 strain, in addition to *bla*_TEM_ and *bla*_SHV_.

On the other hand, although the expression of the *qnr* and *aac(6*′*)Ib-cr* genes would directly contribute to the multi-resistance phenotypes of studied strains, and are often present in CTX-M producing *Enterobacteriaceae*, they are not associated with the genetic platforms where *bla*_CTX-M_ is located. However as these platforms are based on unusual class 1 integrons, they most likely contribute to resistance to other non-assayed antimicrobials, as they encode determinants for resistance to quaternary ammonium compounds (*qacE*Δ*1*), sulphonamides (*Sul1*), trimethoprim (*dfrA12*), and other aminoglycosides (*aadA1, aadA2*).

The high percentage (73%) of resistance to colistin found differs from that described in other geographical areas of the world (Guzmán-Blanco et al., 2014; Pereira et al., 2015; Aires et al., 2017). As such the presence of *mcr-1* and *mcr-2* was assessed by PCR, using primers listed in Tables S2, since these genes were described as the first transferable mechanism of colistin resistance (Liu et al., 2016); none of the isolates was found to carry them. This may be explained by the high genetic identity shared among most strains, suggesting the resistance to colistin may be mediated by chromosomal-encoded mechanisms instead (Liu et al., 2016); it also warrants reconsidering of the use of colistin in the treatment of carbapenemase-producing strains of *K. pneumoniae* in Chile.

The ability of the bacteria to acquire and disseminate exogenous genes across different genetic platforms and mobile genetic elements is one of the major factors involved in the development of multi-drug resistance in the past 50 years (Cantón et al., 2012b). The present work reports the existence of complex or unusual class 1 integrons and IS associated with *bla*_CTX-M_, which may grant these platforms the capacity of horizontal mobilization between bacteria of the same or different species, and the dissemination of this ESBL (Cantón et al., 2012b). Additionally we provide the first epidemiological and molecular data on the prevalence of genes of clinical importance associated with resistance to other antimicrobials in strains producing carbapenemases, isolated in Chilean hospitals.

## Author contributions

HB-T: Management of financial support, overall study design, critical revision of manuscript. SC-A: Acquisition of data, analysis and interpretation of data and drafting of manuscript. AV-L, MQ-A, and MM-R: Experimental work and critical revision of manuscript. CL: Acquisition of data and critical revision of manuscript. JF: Performing and Analysis of MLST sequences. SU: Performing and analysis of PFEG. MD: Collection of bacterial isolates and critical revision of manuscript. GG-R: Design of molecular experiment and critical revision of manuscript.

### Conflict of interest statement

The authors declare that the research was conducted in the absence of any commercial or financial relationships that could be construed as a potential conflict of interest.

## Footnotes

^1^http://bigsdb.pasteur.fr/

^2^https://www.lahey.org/Studies/

^3^https://blast.ncbi.nlm.nih.gov/Blast.cgi

^4^http://web.expasy.org/translate/

^5^http://www.ebi.ac.uk/tools/msa/clustalo/

^6^http://doua.prabi.fr/software/cap3
