# Supplementary material for: Genetic Platforms of blaCTX-M in Carbapenemase-Producing Strains of K. pneumoniae Isolated in Chile
Source: Front Microbiol. 2018 Mar 6;9:324. doi: 10.3389/fmicb.2018.00324 (PMC5857710; doi:10.3389/fmicb.2018.00324)
Supplement: Supplementary file 1 [file Table1.doc]

**Table S1.** PCR primers used for the screening and determination of allele variants of different β-lactamase genes

| Gene target | Primers | Sequence ( 5' --> 3' ) | Size (bp) | Reference |
| --- | --- | --- | --- | --- |
| *bla*_CTX-M-1_ | mCTX-MG1R | AGCTTATTCATCGCCACGTT | 415 | Woodford et al., 2006 |
|  | mCTX-MG1F | AAAAATCACTGCGCCAGTTC |  |  |
| *bla*_CTX-M-2_ | mCTX-MG2R | CCAGCGTCAGATTTTTCAGG | 552 |  |
|  | mCTX-MG2F | CGACGCTACCCCTGCTAT |  |  |
| *bla*_CTX-M-8_ | mCTX-MG8R | AACCCACGATGTGGGTAGC | 666 |  |
|  | mCTX-MG8F | TCGCGTTAAGCGGATGATGC |  |  |
| *bla*_CTX-M-9_ | mCTX-MG9R | AACCCACGATGTGGGTAGC | 205 |  |
|  | mCTX-MG9F | CAAAGAGAGTGCAACGGATG |  |  |
| *bla*_CTX-M-25_ | mCTX-MG25R | AACCCACGATGTGGGTAGC | 327 |  |
|  | mCTX-MG25F | GCACGATGACATTCGGG |  |  |
| *bla*_KPC_ | KPC-F | CGTCTAGTTCTGCTGTCTTG | 798 | Poirel et al., 2011 |
|  | KPC-R | CTTGTCATCCTTGTTAGGCG |  |  |
| *bla*_OXA-48_ | OXA-F | GCGTGGTTAAGGATGAACAC | 438 |  |
|  | OXA-R | CATCAAGTTCAACCCAACCG |  |  |
| *bla_NDM_* | NDM-F | GGTTTGGCGATCTGGTTTTC | 621 |  |
|  | NDM-R | CGGAATGGCTCATCACGATC |  |  |
| *bla*_CTX-M-1_ extragenic | Full CTX-M-F | AAACACACGTGGAATTTAGGG | 820 | Geser et al., 2012 |
|  | CTX-MB | ACCGCGATATCGTTGGT |  |  |
|  | Full CTX-M-R | CCGATGACTATGCGCACTGGG | 828 |  |
|  | CTX-MA | CGCTTTGCGATGTGCAG |  |  |
| *bla*_CTX-M-2_  extragenic | BLA1 | TTAATGATGACTCAGAGCATT | 910 | Vignoli et al., 2006 |
|  | BLA2 | GATACCTCGCTCCATTTATTGC |  |  |
| *bla*_TEM_ | TEM321 | TGGGTGCACGAGTGGGTTAC | 526 | Sánchez et al., 2006 |
|  | TEM834 | TTATCCGCCTCCATCCAGTC |  |  |
| *bla*_SHV_ | SHV1 | GCCCGGGTTATTCTTATTTGTCGC | 1018 |  |
|  | SHV2 | TCTTTCCGATGCCGCCGCCAGTCA |  |  |

## References

1. Geser, N., Stephan, R., Hächler, H. (2012). Ocurrence and characteristics of extended-spectrum β-lactamase (ESBL) producing *Enterobacteriaceae* in food producing animals, minced meat and raw milk. BMC Vet Res. Mar 7; 8: 21. doi: 10.1186/1746-6148-8-21.
2. Poirel, L., Walsh, TR., Cuvillier, V., Nordmann, P. (2011). Multiplex PCR for detection of acquired carbapenemase genes. Diagn Microbiol Infect Dis. 70(1): 119-23. doi: 10.1016/j.diagmicrobio.2010.12.002.
3. Sánchez, M., Bello, H., Domínguez, M., Mella, S., Zemelman, R., González, G. (2006). Transference of extended-spectrum ß-lactamases from nosocomial strains of *Klebsiella pneumoniae* to other species of *Enterobacteriaceae.* Rev Méd Chile; 134: 415-20. doi: 10.4067/S0034-98872006000400002.
4. Vignoli, R., Cordeiro, N., Seija, V., Schelotto, F., Radice, M., Ayala, J.A., Power, P., Gutkind, G. (2006). Genetic environment of CTX-M-2 in *Klebsiella pneumoniae* isolates from hospitalized patients in Uruguay. Rev Argent Microbiol 38: 84-88.
5. Woodford, N., Fagan, E.J., Ellington, MJ. (2006). Multiplex PCR for rapid detection of genes encoding CTX-M extended-spectrum β-lactamases. J Antimicrob Chemother. Jan; 57:154-155. doi: 10.1093/jac/dki412.
