# Supplementary material for: Genetic Platforms of blaCTX-M in Carbapenemase-Producing Strains of K. pneumoniae Isolated in Chile
Source: Front Microbiol. 2018 Mar 6;9:324. doi: 10.3389/fmicb.2018.00324 (PMC5857710; doi:10.3389/fmicb.2018.00324)
Supplement: Supplementary file 2 [file Table2.doc]

**Table S2.** PCR primers used in the screening of different antibiotic resistance genes

| Gene target | Primers | Sequence ( 5' --> 3' ) | Size (bp) | Reference |
| --- | --- | --- | --- | --- |
| *qnrA* | qnrA-F | AGAGGATTTCTCACGCCAGG | 619 | Chen et al., 2012 |
|  | qnrA-R | GCAGCACTATKACTCCCAAGG |  |  |
| *qnrB* | qnrB-F | GGMATHGAAATTCGCCACTG* | 264 |  |
|  | qnrB-r | TTTGCYGYYCGCCAGTCGAA* |  |  |
| *qnrS* | qnrS-F | GCAAGTTCATTGAACAGGCT | 428 |  |
|  | qnrS-R | TCTAAACCGTCGAGTTCGGCG |  |  |
| *qnrC* | qnrC-F | GGGTTGTACATTTATTGAATC | 447 |  |
|  | qnrC-R | TCCACTTTACGAGGTTCT |  |  |
| *qnrD* | qnrC-F | CGAGATCAATTTACGGGGAATA | 582 |  |
|  | qnrC-R | AACAAGCTGAAGCGCCTG |  |  |
| *aac(6')-Ib* | aac(6')-IbF | TTGCGATGCTCTATGAGTGGCTA | 482 |  |
|  | aac(6')-IbR | CTCGAATGCCTGGCGTGTTT |  |  |
| *mcr-1* | crl5-F | CGGTCAGTCCGTTTGTTC | 309 | Liu et al., 2016 |
|  | crl5-R | CTTGGTCGGTCTGTA GGG |  |  |
| *mcr-1* and *mcr-2* | mcr_s_F | TGTTCGTCGTCGGTGAGACGG | 216 | This work |
|  | mcr_s_R | GCACATTTTCTTGGTATTTGG |  |  |

## References

1. Chen, X., Zhang, W., Pan, W., Yin, J., Pan, Z., Gao, S., Jiao, X. (2012). Prevalence of *qnr*, *aac(6′)-Ib-cr, qepA,* and *oqxAB* in *Escherichia coli* isolates from humans, animals, and the environment. Antimicrob. Agents Chemother. Jun; 56(6): 3423-3427. doi: 10.1128/AAC.06191-11.
2. Liu, Y.Y., Wang, Y., Walsh, T.R., Yi, L.X., Zhang, R., Spencer, J., Doi, Y., Tian, G., Dong B., Huang, X., Yu, L.F., Gu, D., Ren, H., Chen, X., Lv, L., He, D., Zhou, H., Liang, Z., Liu, J.H., Shen, J. (2016). Emergence of plasmid-mediated colistin resistance mechanism MCR-1 in animals and human beings in China: a microbiological and molecular biological study. Lancet Infect Dis. 16(2):161-8. doi: 10.1016/S1473-3099(15)00424-7.
