# Supplementary material for: Genetic Platforms of blaCTX-M in Carbapenemase-Producing Strains of K. pneumoniae Isolated in Chile
Source: Front Microbiol. 2018 Mar 6;9:324. doi: 10.3389/fmicb.2018.00324 (PMC5857710; doi:10.3389/fmicb.2018.00324)
Supplement: Supplementary file 3 [file Table3.doc]

**Table S3.** PCR primers used for the determination of the genetic platforms of *bla*CTX-M-2

| Letter | Gen target | Primer | Sequence ( 5' --> 3' ) | Size (pb) | Reference |
| --- | --- | --- | --- | --- | --- |
| A | *intl1* | IntA | GTCAAGGTTCTGGACCAGTTGC | 893 | Gaze et al., 2005 |
|  |  | IntB | ATCATCGTCGTAGAGACGTCGG |  |  |
| B | VR integrón clase 1 | 5’ CS | GGCATCCAAGCAGCAAGC | * | Vignoli et al., 2006 |
|  |  | 3’ CS | AAGCAGACTTGACCTGAT |  |  |
| C | *sul1* | Int-2 | TAGAGCGCAGGGTCAGGAAA | 652 |  |
|  | *qacE*∆*1* | qac. F | ATCGCAATAGTTGGCGAAGT |  |  |
| D | *qacE*∆*1* | qac. F | ATCGCAATAGTTGGCGAAGT | 226 |  |
|  |  | qac. R | CAAGCTTTTGCCCATGAAGC |  |  |
| E | *sul1+orf513* | F12.F | GTATTGCGCCGCTCTTAGAC | 1974 |  |
|  |  | F12.R | AAACCAGCATGGTTGGCTAC |  |  |
| F | *orf513 + bla*_CTX-M-2_ | Orf513InK13 | CACCCTGCAAACCTTGCCAGG | 1309 |  |
|  |  | Blaup | GGCTTCCAGCTGCTGTTGCAC |  |  |
| G | *bla*_CTX-M-2_ | Bla1 | TTAATGATGACTCAGAGCATT | 2212 | Vignoli et al., 2006 |
|  | *qacE*∆*1* | SC2 | CAAGCTTTTGCCCATGAAGC |  | This study |
| H | *Orf3* | SC3 | GAGGGGACAATGAAGTCGCT | 1452 | This study |
|  | *sul1* | SC6 | CGAAGAACCGCACAATCTCG |  |  |
| I | *gcuF/aadA* | SC4 | ATCATGAGGGAAGCGGTGAC | 2454 | This study |
|  | *ISCR-1* | SC5 | TAACCCGCAATAGCCCTCAC |  |  |
|  | *aar2* | aar2 F | CAACGATGTTACGCAGCAGG | * | This study |
|  | *cmla5* | cmla5-F | TGCTCCAATGTGGCTAGTGG |  |  |
|  | IS*CR1* | ISCR1-R | TATTCTCGGCATCACGCTCC |  |  |
|  | *aar-2* | arr2R | GGAGGGGCATCCTGTTGAAT |  |  |
|  | *aadA1* | aadA1-R | AGGCTTGATGAAACAACGCG |  |  |

VR: variable region

*: Variable or used in association with another primer

## References

1. Gaze, W.H., Abdouslam, N., Hawkey, P.M., Wellington, E.M.H. (2005). Incidence of class 1 integrons in a quaternary ammonium compound-polluted environment. Antimicrob Agents Chemother. May; 49(5): 1802-1807. doi: 10.1128/AAC.49.5.1802-1807.2005
2. Vignoli, R., Cordeiro, N., Seija, V., Schelotto, F., Radice, M., Ayala, J.A., Power, P., Gutkind, G. (2006). Genetic environment of CTX-M-2 in *Klebsiella pneumoniae* isolates from hospitalized patients in Uruguay. Rev Argent Microbiol 38: 84-88
