# Supplementary material for: Genetic Platforms of blaCTX-M in Carbapenemase-Producing Strains of K. pneumoniae Isolated in Chile
Source: Front Microbiol. 2018 Mar 6;9:324. doi: 10.3389/fmicb.2018.00324 (PMC5857710; doi:10.3389/fmicb.2018.00324)
Supplement: Supplementary file 4 [file Table4.doc]

**Table S4.** PCR primers used for the determination of the genetic platforms of *bla*_CTX-M-15_

| Letter | Gen target | Primer | Sequence ( 5' --> 3' ) | Size (pb) | Reference |
| --- | --- | --- | --- | --- | --- |
| J | IS*Ecp1 + bla*_CTX-M-1_ | SC11 | TGCTCTGCGGTCACTTCATT | 1729 | This study |
|  |  | SC12 | GCTAAGCTCAGCCAGTGACA |  |  |
| K | IS*26* | IS26-F | ACTCCACGATTTACCGCTGG | 562 |  |
|  |  | IS26-R | ACTCTGCTTACCAGGCGCATTTC |  |  |
| *L* | *bla*_CTX-M-1_ + ∆*orf477* | CTX-M-1_540 | GCGTGATACCACTTCACCTC | 461 | Iroha et al., 2012 |
|  |  | orf477 | GAAGGAGAACCAGGAACCAC |  |  |
| M | IS*Ecp1* | ISEcp1-F | TTCAAAAAGCATAATCAAAGCC | * |  |
|  |  | ISEcp1-R | CAACCACCTTTCAATCATTTTT |  |  |
| *N* | IS*Ecp1 +*  *bla*_CTX-M-15_ | SC7 | GGACTCTTCAGAATACAGACAGCA | 1021 | This work |
|  |  | SC8 | GCTGTCGCCCAATGCTTTAC |  |  |
| *O* | IS*26 +*  IS*Ecp1* | IS26Ecp1.R | CAAAGTTAGCGATGAGGCAG | 775 | Eckert et al., 2006 |
|  |  | ISEcp1 F | TTCAAAAAGCATAATCAAAGCC |  |  |
| *P* | IS*Ecp1* | ISEcp1_CTX_F | TGCTCTGCGGTCACTTCATT | 1991 | This work and Eckert et al., 2006 |
|  |  | ISEcp1 R | CAACCACCTTTCAATCATTTTT |  |  |
| *Q* | ISE*cp1* +  *bla*_CTX-M_ | SC9 | TTTGGGCGAATGAAGCCGT | 1309 | This work |
|  |  | SC10 | CGGCTTTCTGCCTTAGGTTG |  |  |

*: Variable size.

## References

1. Eckert, C., Gautier, V., Arlet, G. (2006). DNA sequence analysis of the genetic environment of various *bla*_CTX-M_ genes. J. Antimicrob Chemother. 57(1): 14-23. doi: 10.1093/jac/dki398.
2. Iroha, I., Esimone, C., Neumann, S., Marlinghaus, L., Korte, M., Szabados, F., Gatermann, S., Kaase M. (2012). First description of *Escherichia coli* producing CTX-M-15- extended spectrum beta lactamase (ESBL) in out-patients from south eastern Nigeria. Ann Clin Microbiol Antimicrob 23; 11:19.
